# AA genotype of *PLIN1* 13041A>G as an unfavourable predictive factor of malnutrition associated with fat mass loss in locally advanced head and neck cancer male patients treated with radiotherapy

**DOI:** 10.1007/s00520-020-05675-8

**Published:** 2020-08-15

**Authors:** Tomasz Powrózek, Anna Brzozowska, Marcin Mazurek, Monika Prendecka, Iwona Homa-Mlak, Radosław Mlak, Teresa Małecka-Massalska

**Affiliations:** 1 Department of Human Physiology, Medical University of Lublin, Lublin, Poland; 2 St. John of Dukla Lublin Region Cancer Center, Lublin, Poland

**Keywords:** Head and neck cancer, Malnutrition, Radiotherapy, PLIN1, Fat mass loss

## Abstract

**Introduction:**

Malnutrition is a frequently diagnosed condition in head and neck cancer (HNC) patients after radiation therapy (RTH). Malnutrition causes adipose tissue dysfunction associated with intensified lipolysis and disruption of the activity of mechanisms that protect adipose tissue against this process, which include the protective function of perilipin.

**Material and methods:**

The purpose of this study was the evaluation of the predictive value of 13041A>G *PLIN1* polymorphism in the development of malnutrition related to adipose tissue loss in a group of 80 patients with locally advanced HNC treated by means of radical radiation therapy.

**Results:**

After the completion of RTH, men with AA genotype had significantly lower fat mass (FM compared to men with G haplotype; FM: 13.84 ± 6.36 kg and 19.06 ± 6.30 kg (*p* = 0.009). In consequence of RTH, the AA genotype carriers lost an average of 37.01% adipose tissue mass and patients with GA and GG genotypes lost 12.82 and 0.31% (*p* = 0.035), respectively. AA genotype was also associated with higher chance of ≥ 10%, ≥ 20% and ≥ 30% FM loss in the course of RTH (OR = 13.78; 5.78; 2.28).

**Conclusions:**

The evaluation of such molecular factors as SNP 13041A>G may have higher predictive value in the development of malnutrition associated with severe loss of fat mass than the subjective scales, e.g., SGA and NRS-2002. The presence of AA genotype on men with HNC before RTH may facilitate earlier nutritional intervention and supportive treatment aimed at limiting or preventing body mass and fat mass loss during the applied treatment.

**Electronic supplementary material:**

The online version of this article (10.1007/s00520-020-05675-8) contains supplementary material, which is available to authorized users.

## Introduction

According to the European Society for Clinical Nutrition and Metabolism (ESPEN), malnutrition is a state resulting from lack of intake or uptake of nutrition that leads to altered body composition and body cell mass leading to diminished physical and mental function and impaired clinical outcome from disease [1]. It is estimated that this condition may concern 3–52% head and neck cancer (HNC) patients at the time of disease diagnosis. This is related to the anatomical location of the tumour at the border of the respiratory and digestive systems, its stage and the degree of the host metabolism disturbance leading to the acquisition of nutrients necessary for further development and expansion of the cancer process [2]. Standard therapeutic options for HNC include surgery, chemotherapy and radiotherapy (RTH) or a combination of these methods. RTH or radiochemotherapy (RCTH) are characterized by high aggressiveness in the destruction of tumour tissue. Unfortunately, they also damage healthy tissues, which results in either the development of malnutrition or intensification of the already existing malnutrition leading to cachexia [3, 4]. The negative influence of the therapy on the nutritional status of HNC patients is confirmed by a high incidence of malnutrition (44–88%) observed after the treatment completion in this group of patients and the toxicity of the therapy leading to digestive disorders such as vomiting, diarrhoea, xerostomia, oral mucositis, taste disorder and loss of appetite [2, 5, 6]. These side effects of the treatment lead to a significant reduction in the supply of food and energy. This promotes the intensification of catabolic processes within the organism, which results in gradual loss of body mass and its remodelling (changes in body composition) associated with progressive proteolysis and/or lipolysis of muscle and/or fat tissue [7–9].

Fat tissue provides the main and dynamic storage of energy substances in the body—in case of their surplus they are stored in fat tissue, but in case of their deficiency they are released from it and consumed depending on the systemic needs. It is also an important metabolic and endocrine centre of the body. In the course of malnutrition and cancer cachexia, the adipose tissue dysfunction is observed, associated with limited anabolism and increased cellular catabolism as a result of the action of substances released by the tumour and host organism. In the course of malnutrition and cachexia, the processes of both lipogenesis and conversion of white fat into brown fat, accompanied by its further lipolysis, are observed [10–12]. In the course of metabolic disorders resulting from the neoplastic process, there is also a disturbance of protective mechanisms preventing lipolysis, which include the activity of perilipin. Perilipin is encoded by *PLIN1* gene and located in adipocytes of white and brown adipose tissue. Its main task in the state of energy balance of the body is to protect fat drops with triglycerides (TG) stored inside them from the access of HSL and ATGL lipases, and thus regulating the process of fat storage and decomposition. This function can be modulated depending on the energy needs, i.e. in states of satiation and energy surplus, the lipase access to TG is inhibited; while in states of starvation and catabolism, perilipin supports TG breakdown by lipases [13–15]. This underlines the potential contribution of perilipin to fat metabolism in conditions such as malnutrition and cachexia, accompanied by strong catabolism. The function of perilipin can be regulated by single nucleotide polymorphisms (SNPs) present in the gene that encodes it. To date, several studies have confirmed the association of *PLIN1* SNPs with the risk of obesity, insulin resistance, diabetes mellitus, higher body weight and hypertension [16]. Among SNPs, rs2304795 (13041A>G) located in the 3′ region of the *PLIN1* gene seems to have a significant role in the regulation of perilipin function and adipose tissue turnover in the body, as its close association with BMI, body weight and obesity risk has been reported [17].

So far, there have been no data available in the literature regarding the influence of *PLIN1* polymorphisms on the risk of the development of malnutrition and cachexia in malignant tumours, including HNC. Since HNC patients belong to a group with a high risk of malnutrition and metabolic disorders associated with fat tissue function, the evaluation of *PLIN1* gene status seems to have a potential predictive value for the development of malnutrition accompanied by a significant loss of fat mass (FM). The purpose of this study was the evaluation of the influence of 13041A>G *PLIN1* polymorphism on the risk of the development of malnutrition related to adipose tissue loss in the course of treatment of patients with locally advanced HNC by means of radical RTH.

## Materials and methods

### Study group

Eighty patients (60 men and 20 women) with locally advanced squamous-cell HNC were included in this study. Among the HNC subtypes, laryngeal tumours (55% of the study group) and stage IV tumours (76.2% of the study group) were predominant. The patients were diagnosed and treated in the Department of Oncology of the Medical University of Lublin between 2015 and 2019. Patients with the history of surgical treatment accounted for 60% of the study group. The study group characteristics and the parameter values assessed before RTH are presented in Table 1. In order to qualify patients for the study protocol, we applied the appropriate inclusion and exclusion criteria. The following inclusion criteria were used: (1) age > 18 years; (2) performance status ≤ 1 according to ECOG-WHO scale; (3) confirmed diagnosis of HNC in stages III–IV; (4) patients who completed full course of RTH; (5) patients whose health status enabled them to grant their written informed consent to participate in the study. The following exclusion criteria were used: (1) patients with pacemakers or cardioverter-defibrillators; (2) patients with amputated limbs or with prostheses or metal implants; (3) the presence of chronic kidney, heart or liver diseases; (4) abnormal plasma sodium and potassium concentrations.

**Table 1:** Baseline characteristics of the study group

Factor		Study group (*n* = 80)
Gender	Male	60 (75%)
	Female	20 (25%)
Age, mean (range)		63 ± 9 (42–87)
	≥ 63	42 (52.5%)
	< 63	38 (47.5%)
Histopathological diagnosis	Squamous cell carcinoma	80 (100%)
Tumour location	Larynx	44 (55%)
	Oropharynx	36 (45%)
T stage	2	12 (14.9%)
	3	23 (28.8%)
	4	45 (56.3%)
N stage	0	18 (22.5%)
	1	11 (13.8%)
	2	44 (55%)
	3	7 (8.7%)
Disease stage	III	19 (23.8%)
	IVA	50 (62.5%)
	IVB	5 (6%)
	IVC	6 (7.7%)
Performance status (PS)	0	61 (76.3%)
	1	19 (23.7%)
Prior surgical treatment	Yes	48 (60%)
	No	32 (40%)
Type of treatment	RTH alone	41 (51.3%)
	Concurrent CRTH	39 (48.7%)
Alcohol consumption	Yes	30 (37.5%)
	No	50 (62.5%)
Smoking status	Smoker	67 (83.8%)
	Non-smoker	13 (16.2%)
	Current smoker	56 (83.6%)
	Former smoker	11 (16.4%)
Weight (kg) male	Mean ± SD	67 ± 12
Weight (kg) female	Mean ± SD	61 ± 11
BMI male	Mean ± SD	23.36 ± 4.43
	≥ 18.5 < 18.5	49 (81.7%) 11 (18.3%)
BMI female	Mean ± SD	22.16 ± 4.22
	≥ 18.5 < 18.5	16 (80%) 4 (20%)
SGA	A	20 (25%)
	B	35 (43.8%)
	C	25 (31.2%)
NRS	2	57 (71.3%)
	3	20 (25%)
	4	3 (3.7%)
Total protein (g/L)	Mean ± SD	6.65 ± 0.54
Albumin (g/L)	Mean ± SD	3.38 ± 0.26
Prealbumin(g/dL)	Mean ± SD	0.24 ± 0.1
Transferrin (g/L)	Mean ± SD	2.50 ± 0.53
TNF-α (pg/mL)	Mean ± SD	9.92 ± 1.56

The studies were approved by the Bioethical Committee of the Medical University in Lublin (no of consent: KE-0254/232/2014). Written informed consent to participate in the study was obtained from each patient.

### Radiotherapy

All patients qualified for the study were treated with radical RTH (7 cycles of treatment) using ONCOR linear accelerator (Siemens). The Intensity Modulated Radiation Therapy (IMRT) technique was used to administer a total dose of 66–70 Gy (daily dose 2 Gy) in 35 fractions. Patients who previously underwent surgical treatment received 66 Gy in 33 fractions for high risk volume, the intermediate and low risk subclinical volumes received 60 Gy and 54 Gy, respectively. Patients undergoing concomitant chemotherapy received from 1 to 4 courses of cisplatin and 5-fluorouracil (PF).

### Nutritional assessment

The values of anthropometric parameters such as body weight and body mass index (BMI) as well as parameters obtained from electrical bioimpedance analysis (BIA) reflecting body composition, i.e. fat mass (FM) and fat free mass (FFM), were evaluated at two time points, before and after RTH. In addition, subjective nutritional status was assessed using Subjective Global Assessment (SGA), while Nutrition Risk Score (NRS-2002) was used to assess the risk of malnutrition development in HNC patients. Next, the values of the examined parameters were compared in order to evaluate their changes after the treatment completion, quantitative and percentage weight loss, BMI and FM were assessed.

BIA was performed using ImpediMed SFB7 BioImp v1.55 bioimpedance analysis device (Pinkenba, QLD, Australia). The same examination conditions were provided for each patient. BIA was performed in the morning, in fasting condition and in supine position with straightened limbs apart from each other and not in contact with the rest of the body. The electrodes were placed on the right side of the patient’s body. Prior to BIA, patients laid down for several minutes to stabilize and balance their body fluids. The measurements were repeated three times, and the results were averaged. FM and fat-free mass (FFM) values obtained by means of BIA were used in the study.

### PLIN1genotyping

DNA was isolated from whole blood samples collected on EDTA using DNA Blood Mini Kit (Qiagen, Canada). Genotyping was performed using real-time PCR, commercially available fluorescent TaqMan SNP genotyping assay (ThermoFisherScientific, USA) targeting the studied SNP and allele discriminating software. DNA amplification was performed using Genotyping Master Mix (ThermoFisherScientific, USA) kit in StepOnePlus Real-Time PCR System (Applied Biosystems, USA). All steps in the determination of the SNP, from isolation to data analysis, were performed according to the protocols provided by the reagent manufacturer.

### Statistical analysis

The MedCalc software version 15.8 (MedCalc software, Belgium) was applied to statistical analysis and graph generation. Data distribution was tested by Shapiro-Wilk method. Based on the test result, all of the studied parameters demonstrated normal distribution of the data; hence, we used parametrical tests in our calculations. Differences in the value of studied parameters among patients with different genotype distribution were tested by one-way analysis of variance (ANOVA test) as well as the Student’s *t* test compared data difference between the two groups. Frequency of the *PLIN1* genotype depending on the clinical-demographic features of the patients was tested by chi-square and Fisher’s exact test. Impact of the studied clinical-demographic and nutritional parameters on the chance of the FM reduction during the RTH was analysed by univariate and multivariate logistic regression model with the odds ratio calculation and corresponding 95% confidence interval (OR and 95% CI). The results presenting *p* values below 0.05 were considered as statistically significant.

## Results

The following genotype distribution of the studied *PLIN1* gene polymorphism was obtained in the study group: AA in 29 patients (36.2%), GA in 37 patients (46.3%) and GG in 14 cases (17.5%). The distribution of genotypes was consistent with the Hardy-Weinberg’s equilibrium (HWE) in the entire study group (*p* = 0.712) as well as among men (*p* = 0.757) and women (*p* = 0.858). The distribution of SNP genotypes did not depend on the clinical-demographic features of HNC patients, such as age, gender, disease stage and anatomical location of the tumour or the treatment applied before RTH. There were no significant differences in the studied parameters between patients previously treated surgically (*p* > 0.05) (Supplementary Table 1).

**Fig. 1:**
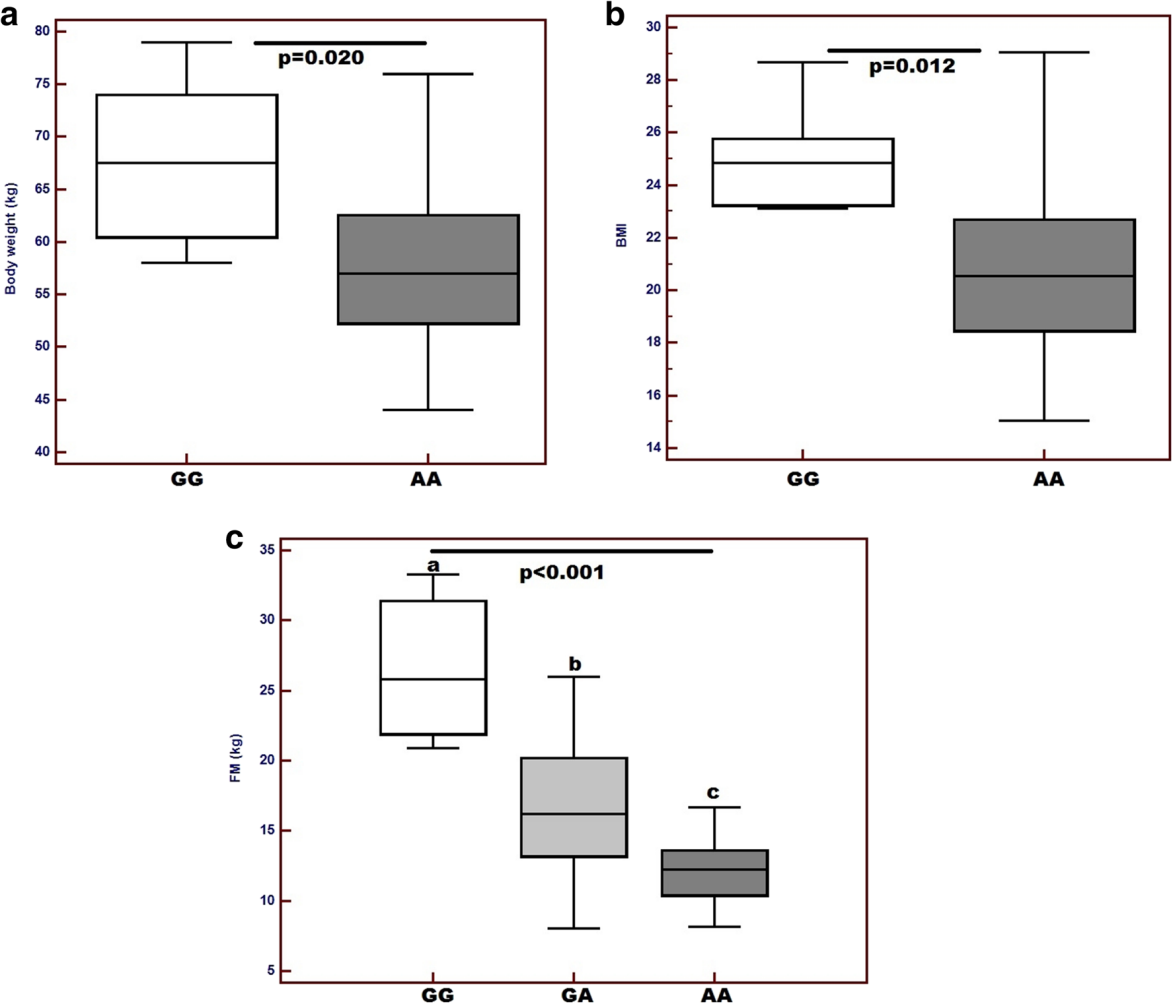
Differences in the anthropometrical parameters and fat mass (FM) between group of male patients carrying various *PLIN1* genotypes—measurements after the RTH: **a** comparison of the body weight between AA and GG homozygous patients; **b** differences in BMI between AA and GG homozygous patients, and **c** comparison of FM values among all PLIN1 genotype carriers

The differences in the anthropometric parameter values (body weight, BMI) and body composition parameters (FM and FFM) in relation to the genotype before and after RTH are presented in Table 2. Significant differences in BMI and FM were observed in the group of men before and after RTH. AA genotype carriers had significantly lower BMI than GA and GG genotypes (mean BMI: 22.61 ± 4.64 and 22.75 ± 4.09 and 27.12 ± 2.45, respectively; *p* = 0.034). Men with AA genotype also had significantly lower FM compared with the carriers of other genotypes (mean FM: 18.73 ± 7.69 kg for AA and 19.60 ± 7.08 kg and 26.84 ± 4.50 kg for GA and GG, respectively; *p* = 0.037). A similar relationship was observed in A allele carriers compared with GG genotype. At the end of 7 RTH cycles, significant changes in both anthropometric and body composition parameters were observed. Similarly to the measurements performed before the initiation of treatment, significant differences were observed only in men. After the completion of RTH, HNC patients with A haplotype had significantly lower body weight, BMI value and FM compared with men with GG genotype: mean body weight: 59.15 ± 8.98 kg and 68.43 ± 8.02 kg (*p* = 0.014), mean BMI: 20.71 ± 3.29 and 25.19 ± 1.88 (*p* = 0.011) as well as mean FM: 15.50 ± 5.49 kg and 26.76 ± 5.18 kg (*p* < 0.001). Comparing only homozygous patients, men with AA genotype had significantly lower body weight (*p* = 0.020; Fig. 1a) and BMI (*p* = 0.012; Fig. 1b) in contrast to GG genotype carriers. Moreover, AA genotype carriers had significantly lower FM values compared with G allele carriers (GG and GA): FM FM 13.84 ± 6.36 kg and 19.06 ± 6.30 kg (*p* = 0.009) (Fig. 1c).

**Table 2:** The differences in the anthropometric parameter values (body weight, BMI) and body composition parameters (FM and FFM) in relation to the genotype before and after RTH

Factor	GG	GA	AA	*p*	AA	GA + GG	*p*	GG	GA + AA	*p*
Measurements prior to RTH										
Weight (kg) group	70.19 ± 11.21	65.92 ± 11.12	64.00 ± 12.83	0.376	64.00 ± 12.83	67.19 ± 11.17	0.326	70.19 ± 11.21	65.06 ± 11.82	0.198
Weight (kg) men	72.11 ± 11.65	65.60 ± 11.29	66.47 ± 12.13	0.616	66.47 ± 12.13	67.50 ± 11.37	0.722	72.11 ± 11.65	65.98 ± 11.51	0.102
Weight (kg) women	64.0 ± 11.79	66.75 ± 9.98	53.20 ± 9.96	0.173	53.20 ± 9.96	65.86 ± 11.0	0.111	64.0 ± 11.79	59.86 ± 13.17	0.617
BMI group	25.47 ± 3.89	22.96 ± 4.43	22.74 ± 4.84	0.227	22.74 ± 4.84	23.71 ± 4.39	0.443	25.47 ± 3.89	22.86 ± 4.57	0.095
BMI men	27.12 ± 2.45	22.75 ± 4.09	22.61 ± 4.64	*0.034*	22.61 ± 4.64	23.77 ± 4.18	0.904	27.12 ± 2.45	23.11 ± 4.29	*0.021*
BMI women	22.61 ± 4.60	24.55 ± 6.22	19.88 ± 4.22	0.405	19.88 ± 4.22	23.44 ± 5.57	0.214	22.61 ± 4.60	21.43 ± 6.14	0.748
FM (kg) group	23.75 ± 11.11	19.52 ± 6.76	17.01 ± 7.84	0.144	17.01 ± 7.84	21.07 ± 8.48	0.077	23.75 ± 11.11	18.40 ± 7.29	0.053
FM (kg) men	26.84 ± 4.50	19.60 ± 7.08	18.73 ± 7.69	*0.037*	18.73 ± 7.69	21.29 ± 7.21	0.258	26.84 ± 4.50	19.23 ± 7.26	*0.011*
FM (kg) women	21.08 ± 14.50	18.88 ± 4.37	9.72 ± 2.50	0.394	9.72 ± 2.50	20.14 ± 13.39	0.166	21.08 ± 14.50	13.65 ± 5.79	0.337
FM (%) group	32.28 ± 9.26	29.05 ± 7.01	25.75 ± 8.64	0.093	25.75 ± 8.64	30.0 ± 7.76	0.060	32.28 ± 9.26	27.58 ± 7.87	0.090
FM (%) men	36.30 ± 4.02	29.28 ± 7.41	27.45 ± 8.65	0.067	27.45 ± 8.65	30.92 ± 7.36	0.155	36.30 ± 4.02	28.51 ± 7.92	*0.015*
FM (%) women	25.24 ± 12.25	27.32 ± 2.16	18.47 ± 3.26	0.330	18.47 ± 3.26	26.13 ± 8.82	0.135	25.24 ± 12.25	22.26 ± 5.41	0.582
FFM (kg) group	49.28 ± 8.26	47.31 ± 7.04	47.92 ± 7.09	0.756	47.92 ± 7.09	47.90 ± 7.36	0.990	49.28 ± 8.26	47.59 ± 7.00	0.487
FFM (kg) men	47.13 ± 5.74	46.98 ± 7.11	47.99 ± 7.70	0.902	47.99 ± 7.70	47.01 ± 6.72	0.650	47.13 ± 5.74	47.41 ± 7.29	0.922
FFM (kg) women	53.06 ± 11.48	49.87 ± 7.32	47.62 ± 4.38	0.668	47.62 ± 4.38	51.69 ± 9.31	0.439	53.06 ± 11.48	48.58 ± 5.38	0.393
Measurements after commencement of RTH										
Weight (kg) group	65.10 ± 10.23	60.04 ± 8.84	56.47 ± 9.31	0.081	56.47 ± 9.31	61.54 ± 9.42	0.053	65.10 ± 10.23	58.45 ± 9.13	*0.038*
Weight (kg) men	68.43 ± 8.02	59.91 ± 8.87	58.12 ± 9.30	*0.042*	58.12 ± 9.30	61.90 ± 9.29	0.187	68.43 ± 8.02	59.15 ± 8.98	*0.014*
Weight (kg) women	59.25 ± 12.15	60.50 ± 8.66	49.80 ± 5.25	0.182	49.80 ± 5.25	60.0 ± 10.57	0.105	59.25 ± 12.15	54.43 ± 9.62	0.484
BMI group	23.64 ± 3.64	20.97 ± 3.76	20.00 ± 3.18	0.064	20.00 ± 3.18	21.76 ± 4.05	0.093	23.64 ± 3.64	20.54 ± 3.65	*0.014*
BMI men	25.19 ± 1.88	20.82 ± 3.52	20.56 ± 3.06	*0.005*	20.56 ± 3.06	21.84 ± 3.70	0.233	25.19 ± 1.88	20.71 ± 3.29	*0.011*
BMI women	20.93 ± 4.67	22.41 ± 6.47	18.65 ± 3.33	0.524	18.65 ± 3.33	21.44 ± 5.67	0.249	20.93 ± 4.67	19.56 ± 5.52	0.687
FM (kg) group	23.45 ± 10.89	16.79 ± 4.74	13.42 ± 5.77	*< 0.001*	13.42 ± 5.77	19.07 ± 7.82	*0.006*	23.45 ± 10.89	15.28 ± 5.43	*< 0.001*
FM (kg) men	26.76 ± 5.18	16.71 ± 4.51	13.84 ± 6.36	*< 0.001*	13.84 ± 6.36	19.06 ± 6.30	*0.009*	26.76 ± 5.18	15.50 ± 5.49	*< 0.001*
FM (kg) women	20.40 ± 11.52	17.33 ± 7.47	11.60 ± 1.00	0.258	11.60 ± 1.00	19.08 ± 13.20	0.300	20.40 ± 11.52	14.05 ± 5.33	0.382
FM (%) group	32.53 ± 9.09	27.19 ± 5.22	22.96 ± 6.44	*0.001*	22.96 ± 6.44	28.77 ± 6.93	*0.003*	32.53 ± 9.09	25.30 ± 6.12	*0.002*
FM (%) men	36.51 ± 4.40	27.11 ± 5.17	23.44 ± 6.99	*< 0.001*	23.44 ± 6.99	29.30 ± 6.38	*0.005*	36.51 ± 4.40	25.55 ± 6.21	*< 0.001*
FM (%) women	25.57 ± 11.63	27.78 ± 6.78	20.91 ± 3.03	0.537	20.91 ± 3.03	26.52 ± 9.18	0.275	25.57 ± 11.63	23.86 ± 5.78	0.748
FFM (kg) group	47.76 ± 7.18	44.23 ± 6.72	43.99 ± 7.08	0.302	43.99 ± 7.08	45.28 ± 6.95	0.506	47.76 ± 7.18	44.13 ± 6.81	0.121
FFM (kg) men	46.32 ± 4.75	44.32 ± 7.03	43.90 ± 7.37	0.731	43.90 ± 7.37	44.78 ± 6.55	0.672	46.32 ± 4.75	44.14 ± 7.08	0.440
FFM (kg) women	50.27 ± 10.65	43.57 ± 4.48	44.43 ± 6.68	0.494	44.43 ± 6.68	47.40 ± 8.73	0.574	50.27 ± 10.65	44.07 ± 5.41	0.233

**Fig. 2:**
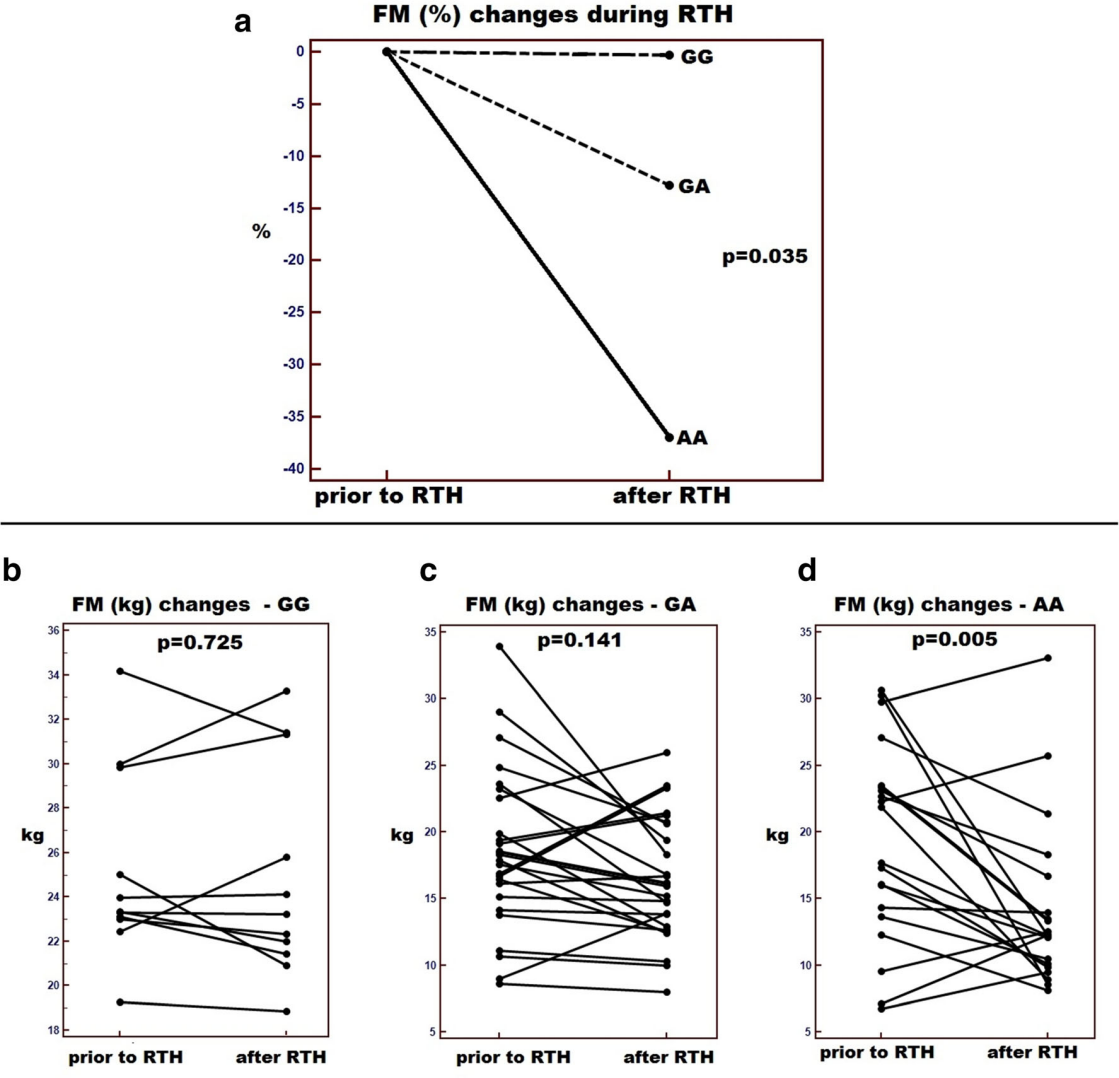
Changes in fat mass (FM) as a consequence of the RTH: **a** percentage change in FM depending on *PLIN1* genotypes; changes in FM as a consequence of the RTH expressed in kg: **b** changes in the group GG carriers; **c** changes in the group GA carriers; **d** changes in the group AA carriers

Since significant changes in the studied parameter values were observed only in the group of men diagnosed with HNC, further analysis was performed in this group of patients. In result of the treatment, 34 men (56.7% of the male subjects) had malnutrition defined as ≥ 5% body weight loss before RTH. The highest percentage of malnourished men was found in the group of AA genotype carriers (76.2%), which was significantly higher than in the case of patients with GA and GG genotypes (53.6% and 27.3%) (*p* = 0.027). With regard to FM in consequence of RTH, the AA genotype carriers lost an average of 37.01% fat mass and patients with GA and GG genotypes lost 12.82 and 0.31% (*p* = 0.035)(Fig. 2a), respectively. After the completion of treatment in the group of patients with GG genotype, the mean FM loss was < 0.1 kg (*p* = 0.725, Fig. 2b), in men with GA genotype the mean loss was 2.89 kg (*p* = 0.141, Fig. 2c) and in carriers of AA genotype 5.88 kg (*p* = 0.005, Fig. 2d). Moreover, it was found that the percentage of men who lost at least 10%, 20% or 30% body weight in result of treatment was the highest in the group of patients with AA genotype. Significant FM loss defined as fat loss of ≥ 20% or ≥ 30% was observed in 52.4% and 38.1% of patients with AA genotype, respectively. These percentages were significantly higher than in carriers of other genotypes (*p* = 0.004 and *p* = 0.037). We did not observed any significant differences concerning FFM values. The differences in the values of the studied anthropometric and FM parameters (before and after RTH) according to *PLIN1* genotype are presented in Table 3.

**Table 3:** Changes in the anthropometrical and nutritional parameters in a consequence of the RTH in different genotype carriers of *PLIN1*

Factor— mean changes after RTH	GG	GA	AA	*p*
Weight	− 4.82%	− 5.0%	− 7.20%	0.284
Weight loss > 5%	3/11 (27.3%)	15/28 (53.6%)	16/21 (76.2%)	*0.027*
BMI	− 7.08%	− 7.96%	− 10.80%	0.387
FM	− 0.31%	− 12.82%	− 37.01%	*0.035*
FM loss (kg)	− 0.10	− 2.89	− 5.88	*0.011*
FM loss > 10%	3/11 (27.3%)	15/28 (53.6%)	16/21 (76.2%)	*0.027*
FM loss > 20%	0/11 (0%)	6/28 (21.4%)	11/21 (52.4%)	*0.004*
FM loss > 30%	0/11 (0%)	5/28 (17.9%)	8/21 (38.1%)	*0.037*
FFM loss (kg)	− 0.81	− 2.66	− 4.09	0.642
Factor—mean changes after RTH	GG and GA		AA	*p*
Weight	− 4.92%		− 7.20%	0.173
Weight loss > 5%	18/39 (46.2%)		16/21 (76.2%)	*0.031*
BMI	− 7.77%		− 10.80%	0.217
FM	− 7.73%		− 37.01%	*0.012*
FM loss (kg)	− 2.23		− 5.88	*0.033*
FM loss > 10%	18/39 (46.2%)		16/21 (76.2%)	*0.031*
FM loss > 20%	6/39 (15.4%)		11/21 (52.4%)	*0.006*
FM loss > 30%	5/39 (12.8%)		8/21 (38.1%)	*0.045*
FFM loss (kg)	− 2.23		− 4.09	0.438

In the last stage of the study, we assessed the factors that significantly affect the chances of FM loss (in three models, respectively: ≥ 10%, ≥ 20% and ≥ 30%) following RTH in the study group of men with HNC. All patient clinical and demographic features, anthropometric parameters, BIA and the treatment applied were included in the analyses. Univariate analysis selected only AA genotype as a predictive factor significantly affecting the risk of FM loss in each of the studied models. Patients with AA genotype were 8, 5 and 2 times more likely to lose ≥ 10%, ≥ 20% and ≥ 30% FM than the carriers of other genotypes, respectively. In the multivariate analysis, which included all the clinical-demographic-nutritional data of the studied men, a significant influence of AA genotype on the chance of FM loss during RTH was also observed. Unfavourable predictive factors of ≥ 10% loss were SGA-C (OR = 3.78, *p* = 0.043) and AA genotype (OR = 13.78, *p* = 0.032), of ≥ 20% loss, it was AA genotype (OR = 5.78, *p* = 0.010) and of ≥ 30% loss, it was also AA genotype (OR = 2.28, *p* = 0.023) (Table 4).

**Table 4:** Factors affecting the chance of fat mass loss during the course of RTH

Factor	Univariate analysis		Multivariate analysis	
	*p*	OR [95%CI]	*p*	OR [95%CI]
loss of FM > 10%				
AA genotype	0.043	8.12 [0.892–43.844]	0.032	13.78 [1.247–152.31]
SGA-C	–	–	0.043	3.78 [0.803–17.844]
loss of FM > 20%				
AA genotype	0.013	5.04 [1.399–18.175]	0.010	5.78 [1.521–21.932]
loss of FM > 30%				
AA genotype	0.018	2.23 [0.687–7.247]	0.028	2.28 [0.892–15.435]

## Discussion

The incidence of malnutrition observed in HNC patients in radiological wards can reach even 80%. The presence of malnutrition is associated with unfavourable prognosis of the disease, higher mortality and deterioration of the quality of life; therefore, it is necessary to identify the patients with high risk of malnutrition and cachexia [18]. Based on the above, the classical definition of malnutrition has been extended and also refers to other factors conducive to the development of malnutrition, apart from those related to tumour presence and systemic metabolic disorders. It is believed that any involuntary weight loss ≥ 5% within 1 month is a reliable indicator of malnutrition associated with hospitalization and treatment [19]. This emphasizes that the therapy and the adverse events associated with it may significantly affect the dynamics of malnutrition development, even in such short periods of time, which are close to the duration of radical RTH (6–8 weeks). Currently used subjective methods of nutrition evaluation such as SGA or NRS and anthropometric parameters such as body weight and BMI may be insufficient to detect malnutrition and malnutrition associated with significant FM loss. Despite the loss of fat mass, the above parameters may be correct, thus masking the development of malnutrition. Therefore, it seems justified to search for molecular predictive markers of malnutrition that reflect the status of the organism at the cellular level.

Some studies show that white adipose tissue is one of the first organs affected by malnutrition or cachexia, the symptom of which is the reduction of its mass, often without visible muscle tissue loss. Increased mobilization of fat tissue in both syndromes is associated with increased activity of ATGL and HSL lipases, whose access to triglycerides stored in fat drops is regulated by perilipin [20]. It has also been noted that the impairment of perilipin function stimulates the development of inflammatory response in adipocytes by secretion of proinflammatory fat metabolites resulting from their uncontrolled breakdown [21]. To date, several SNPs have been identified in the perilipin-coding gene, including rs 1052700, rs 894160, rs 2289487 and rs 2304795 (13041A>G), which are associated with the risk of obesity associated with fat accumulation, increased BMI and body weight [17]. SNP 13041 A > G seems to have a significant influence on perilipin activity due to its genomic location. It is located in the 3′ region of the gene—a place of alternative splicing, where various isoforms of perilipin are formed during PLIN1 transcription, manifesting varied protective activity of fats against their lipolysis [22]. In a study of 734 subjects, Qi et al. noted a higher incidence of the G allele of 13041A>G and its influence on the risk of the development of obesity. The authors confirmed the relationship between SNP and obesity only in the group of women. The average percentage of fat tissue in women was 30.6%, 32.7% and 33.3% in AA, GA and GG genotypes respectively (p = 0.016). Moreover, G allele carriers (GG and GA) had significantly higher BMI and obesity risk (OR = 1.73) than women with AA genotype [23].

In another study conducted by the same authors, in a group of over 4000 subjects of Asian origin, it was observed that the presence of the GG 13041A>G genotype was associated with the risk of obesity approximately twice as high as in patients with GA and AA variants. This trend was observed in the Malaysian population; whereas in the obese individuals of Chinese and Indian origin, the G allele frequency was higher [24]. In another study, Jenkis et al. made similar observations in a group of the obese elderly subjects. Individuals carrying AA genotype had significantly lower BMI and fat content than those carrying other genotypes. Even after 6 months of workout to reduce body weight and body fat, AA genotype carriers reported significantly lower BMI and body fat mass than other individuals [25]. Our findings seem to be consistent with those obtained in those studies, although we only reported statistically significant results for men. Men with A haplotype had significantly lower BMI and FM before the start of treatment compared with GA or GG genotype carriers (*p* = 0.021 and 0.011, respectively). After the RTH the AA men had significantly lower FM compared with G haplotype carriers (*p* = 0.009). Interestingly, we observed that in result of the treatment, men with AA genotype lost a much higher percentage of body fat than the carriers of GA and GG genotypes, with the loss of − 0.31%, − 12.82% and − 37.01% (*p* = 0.035), respectively. The percentage of malnourished patients was also higher in the group of men with AA genotype (76.2%) than in patients with GA and GG genotypes (46.2%) (*p* = 0.031). The presence of AA genotype had a significant influence on the chance of FM loss during RTH; > 10% FM (OR = 13.78), > 20% FM (OR = 5.78) and > 30% FM (OR = 2.28). Few studies did not confirm the relationship between 13041A>G and the body nutritional parameters. Soenenen et al. and Ruiz et al. evaluated the influence of low calorie and low energy diets on body composition parameters in relation to *PLIN1* 13041A>G genotype. They did not report any significant correlations between the genotype variants of the studied SNPs and the body composition parameters in subjects after a 12-week diet [26, 27]. It should be noted, however, that these studies were aimed at the evaluation of the influence of diet on weight reduction in relation to the genotype. To this day, there have been no studies on the influence of *PLIN1* polymorphisms on the nutritional status of malignant tumours and their relation to the treatment used. Perhaps the mechanism associated with the regulation of perilipin activity is completely different in cancer patients because other pathways of lipolysis are additionally activated. Radical RTH may also have a significant influence on the regulation of inflammatory and metabolic response of the organism, hence the genotypic variants of perilipin conditioned by 13041A>G may manifest different protective activity of adipose tissue against uncontrolled lipolysis. The evaluation of such molecular factors as SNP 13041A>G may also have higher predictive value in the development of malnutrition associated with severe loss of fat mass than the subjective scales, e.g. SGA and NRS-2002. NRS scoring did not significantly affect the chance of FM loss during RTH, while the presence of SGA-C was associated only with the chance of FM loss above 10% (OR = 3.78 compared with OR = 13.78 for AA genotype).

Our study is not devoid of some limitations. First of all, it was performed in a relatively small group of patients. In our group, there is also a disproportion in the number of men and women (3:1) as a result of more frequent occurrence of HNC in men. In the quoted studies, the ratio was 1:1 or 1:2, which may have resulted in a lack of correlation between the assessed SNP and the body composition parameters in the studied women. Moreover, we performed the evaluation at two time points, before and after the treatment completion. The evaluation carried out after each RTH cycle could more clearly show the dynamics of changes in body composition. Despite these limitations, it should be noted that this is the first study to evaluate the *PLIN1* 13041A>G value in predicting adverse changes in body composition during RTH. The presence of AA genotype on men with HNC before RTH may facilitate earlier nutritional intervention and supportive treatment aimed at limiting or preventing body mass and fat mass loss during the applied treatment. According to literature data, most HNC patients require nutritional intervention at regular intervals during RTH [28]. The results of our study should be confirmed in larger groups of patients, especially including higher number of women, in order to finally confirm the predictive value of SNP in patients with HNC treated with radical RTH.

## Electronic supplementary material

ESM 1 (DOCX 15 kb)
