# Supplementary material for: AA genotype of PLIN1 13041A>G as an unfavourable predictive factor of malnutrition associated with fat mass loss in locally advanced head and neck cancer male patients treated with radiotherapy
Source: Support Care Cancer. 2020 Aug 15;29(4):1923–32. doi: 10.1007/s00520-020-05675-8 (PMC7892500; doi:10.1007/s00520-020-05675-8)
Supplement: Supplementary file 1 — (DOCX 15 kb) [file 520_2020_5675_MOESM1_ESM.docx]

**Supplementary table 1.** Distribution of the clinical-demographic data among patients with different *PLIN1* genotypes

| **Factor** | | **GG (n=14; 17.5%)** | **GA (n=37;**  **46.3%)** | **AA (n=29;**  **36.2%)** | ***p*** | **AA (n=29; 36.2%)** | **GA+GG (n=51; 63.8%)** | ***p*** | **GG (n=14; 17.5%)** | **GA+AA (n=66; 82.5%)** | ***p*** |
| --- | --- | --- | --- | --- | --- | --- | --- | --- | --- | --- | --- |
| **Gender** | **Male** | 11 (18.3%) | 28 (46.7%) | 21 (35%) | 0.901 | 21 (35%) | 39 (65%) | 0.790 | 11 (18.3%) | 49 (81.7%) | 1.0 |
|  | **Female** | 3 (15%) | 9 (45%) | 8 (40%) |  | 8 (40%) | 12 (60%) |  | 3 (15%) | 17 (85%) |  |
| **Age**  **(mean; years)** | **>63** | 8 (19%) | 19 (45.2%) | 15 (35.8%) | 0.929 | 15 (35.8%) | 27 (64.2%) | 1.0 | 8 (19%) | 34 (81%) | 0.774 |
|  | **<63** | 6 (15.8%) | 18 (47.4%) | 14 (36.8%) |  | 14 (36.8%) | 24 (63.2%) |  | 6 (15.8%) | 32 (84.2%) |  |
| **Tumor location** | **Upper throat** | 5 (18.6%) | 11 (40.7%) | 11 (40.7%) | 0.772 | 11 (40.7%) | 16 (59.3%) | 0.626 | 5 (18.6%) | 22 (81.4%) | 1.0 |
|  | **Lower throat** | 9 (17%) | 26 (49.1%) | 18 (33.9%) |  | 18 (33.9%) | 35 (66.1%) |  | 9 (17%) | 44 (83%) |  |
|  | **Larynx** | 6 (13.6%) | 22 (50%) | 16 (36.4%) | 0.568 | 16 (36.4%) | 28 (63.6%) | 1.0 | 6 (13.6%) | 38 (86.4%) | 0.382 |
|  | **Others** | 8 (22.2%) | 15 (41.7%) | 13 (36.1%) |  | 13 (36.1%) | 23 (63.9%) |  | 8 (22.2%) | 28 (77.8%) |  |
| **Disease stage** | **III** | 4 (21%) | 9 (47.4%) | 6 (31.6%) | 0.845 | 6 (31.6%) | 13 (68.4%) | 0.786 | 4 (21.1%) | 15 (78.9%) | 0.732 |
|  | **IV** | 10 (16.4%) | 28 (45.9%) | 23 (37.7%) |  | 23 (37.7%) | 38 (62.3%) |  | 10 (16.4%) | 51 (83.6%) |  |
| **Tumor T-stage** | **II** | 3 (31%) | 5 (35.7%) | 4 (33.3%) | 0.513 | 4 (33.3%) | 8 (66.7%) | 0.413 | 3 (31%) | 9 (69%) | 0.675 |
|  | **III** | 3 (13%) | 14 (60.9%) | 6 (26.1%) |  | 6 (26.1%) | 17 (73.9%) |  | 3 (13%) | 20 (87%) |  |
|  | **IV** | 8 (17.8%) | 18 (40%) | 19 (42.2%) |  | 19 (42.2%) | 26 (57.8%) |  | 8 (17.8%) | 37 (82.2%) |  |
| **Performance**  **status** | **0** | 12 (19.7%) | 26 (42.6%) | 23 (37.7%) | 0.455 | 23 (37.7%) | 38 (62.3%) | 0.786 | 12 (19.7%) | 49 (80.3%) | 0.500 |
|  | **1** | 2 (10.5%) | 11 (57.9%) | 6 (31.6%) |  | 6 (31.6%) | 13 (68.4%) |  | 2 (10.5%) | 17 (89.5%) |  |
| **Prior surgical**  **treatment** | **Yes** | 9 (18.8%) | 19 (39.6%) | 20 (41.6%) | 0.328 | 20 (41.6%) | 28 (58.4%) | 0.244 | 9 (18.8%) | 39 (81.2%) | 0.773 |
|  | **No** | 5 (15.6%) | 18 (56.3%) | 9 (28.1%) |  | 9 (28.1%) | 23 (71.9%) |  | 5 (15.6%) | 27 (84.4%) |  |
| **Alcohol**  **consumption** | **Yes** | 5 (16.7%) | 17 (56.7%) | 8 (26.6%) | 0.307 | 8 (26.6%) | 22 (73.4%) | 0.231 | 5 (16.7%) | 25 (83.3%) | 1.0 |
|  | **No** | 9 (18%) | 20 (40%) | 21 (42%) |  | 21 (42%) | 29 (58%) |  | 9 (18%) | 41 (82%) |  |
| **Smoking**  **status** | **Smoker** | 11 (16.4%) | 31 (46.3%) | 25 (37.3%) | 0.817 | 25 (37.3%) | 42 (62.7%) | 0.760 | 11 (16.4%) | 56 (83.6%) | 0.690 |
|  | **Non-smoker** | 3 (23.1%) | 6 (46.2%) | 4 (30.7%) |  | 4 (30.7%) | 9 (69.3%) |  | 3 (21.1%) | 10 (78.9%) |  |
| **SGA** | **A** | 5 (25%) | 9 (45%) | 6 (30%) | 0.868 | 14 (70%) | 6 (30%) | 0.759 | 5 (25%) | 15 (75%) | 0.586 |
|  | **B** | 5 (14.3%) | 16 (45.7%) | 14 (40%) |  | 21 (60%) | 14 (40%) |  | 5 (14.3%) | 30 (85.7%) |  |
|  | **C** | 4 (16%) | 12 (48%) | 9 (36%) |  | 16 (64%) | 9 (36%) |  | 4 (16%) | 21 (84%) |  |
| **NRS** | **2** | 12 (21.1%) | 26 (51%) | 19 (27.9%) | 0.658 | 19 (27.9%) | 38 (72.1%) | 0.643 | 12 (21.1%) | 45 (78.9%) | 0.384 |
|  | **3** | 2 (10%) | 9 (45%) | 9 (45%) |  | 9 (45%) | 11 (55%) |  | 2 (10%) | 18 (90%) |  |
|  | **4** | 0 | 2 (66.7%) | 1 (33.3%) |  | 1 (33.3%) | 2 (66.7%) |  | 0 | 3 |  |
